# Supplementary material for: Low-force pulse switching of ferroelectric polarization enabled by imprint field
Source: Nat Commun. 2025 Jun 6;16:5271. doi: 10.1038/s41467-025-60602-x (PMC12144241; doi:10.1038/s41467-025-60602-x)
Supplement: Supplementary file 1 — Supplementary Information [file 41467_2025_60602_MOESM1_ESM.pdf]

Supplementary Information

Low-Force Pulse Switching of Ferroelectric Polarization Enabled by Imprint Field

Yuchao Zhang [1], Shanzheng Du [1], Xiaochi Liu [1], Yahua Yuan [1], Yumei Jing [1], Tian Tian [2,], Junhao Chu [2], Fei Xue [3,4,*], Chang Kai [3], and Jian Sun [1,5,*]*

[1] School of Physics, Central South University, Changsha 410083, China.

[2]Department of Materials Science, Fudan University, Shanghai 200433, China.

[3]Center for Quantum Matter, School of Physics, Zhejiang University, Hangzhou 310027, China.

[4]ZJU-Hangzhou Global Scientific and Technological Innovation Center, Zhejiang University, Hangzhou 311215, China

[5]State Key Laboratory of Powder Metallurgy, Central South University, Changsha 410083, China.

* E-mail: 21110300057@m.fudan.edu.cn and xuef@zju.edu.cn and jian.sun@csu.edu.cn

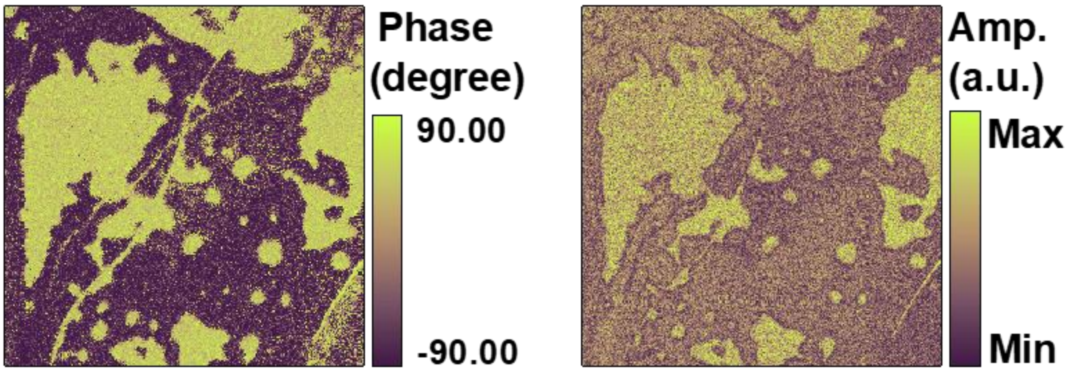

Supplementary Figure 1. Out-of-plane PFM phase and amplitude images of spontaneously polarized CIPS (size: 4 μm×4 μm). Clear domain structures with opposite polarizations can be identified.

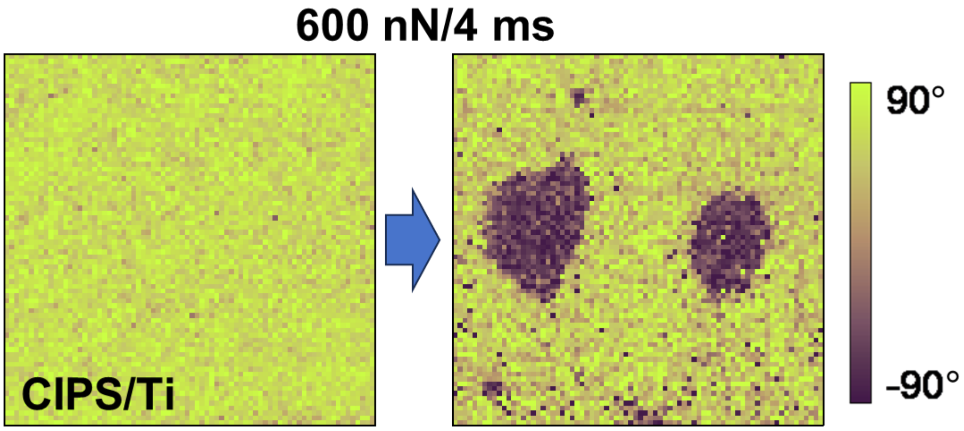

Supplementary Figure 2. Additional measurements of PFM phase images of CIPS on Ti confirming the polarization switching induced by 4 ms-long 600 nN force pulse. Size: 1 μm × 1 μm.

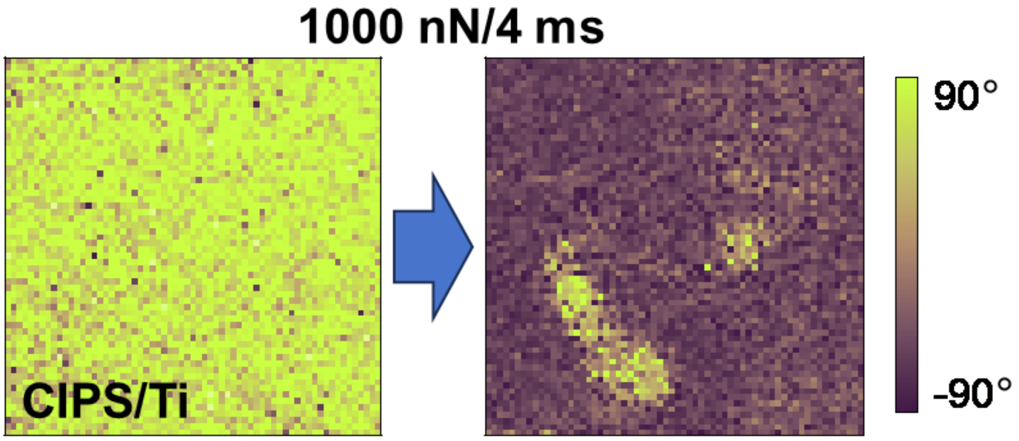

Supplementary Figure 3. PFM phase images of CIPS on Ti measured before and after a 4 ms-long 1000 nN force pulse. Size: 1 μm × 1 μm.

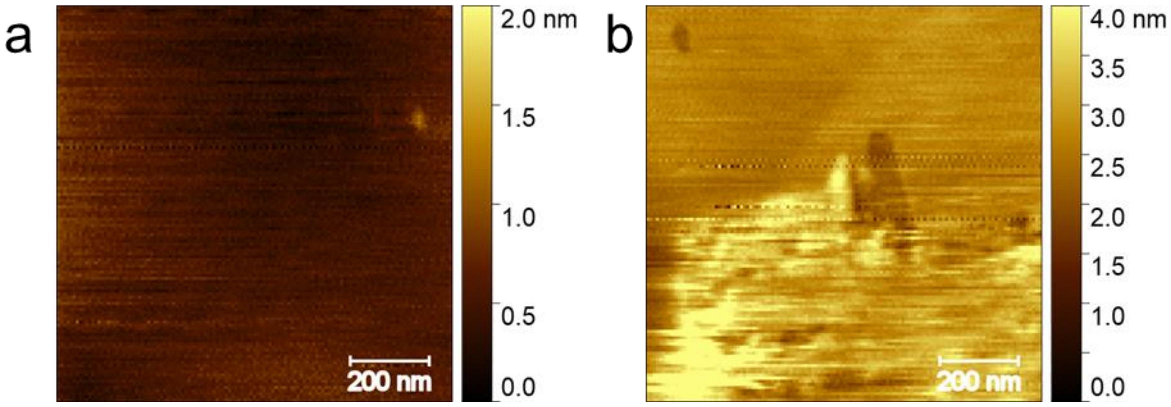

Supplementary Figure 4. AFM topography images of CIPS after different force operations. (a) after multiple 600 nN low force pulses. (b) after multiple 1000 nN high force pulses. Clear damages are observed after 1000 nN high pulses.

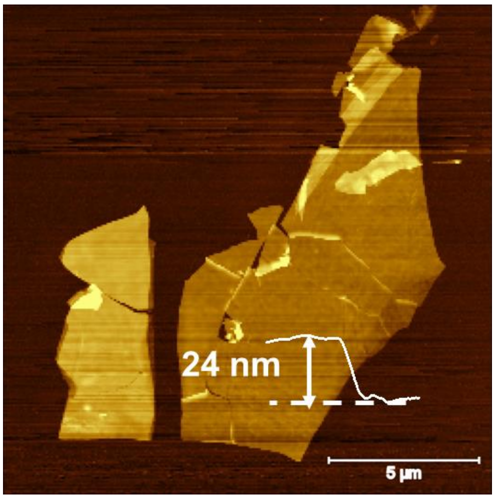

Supplementary Figure 5. AFM images of a thinner CIPS sample on Ti substrate, the line height profile indicates the thickness of ~24 nm.

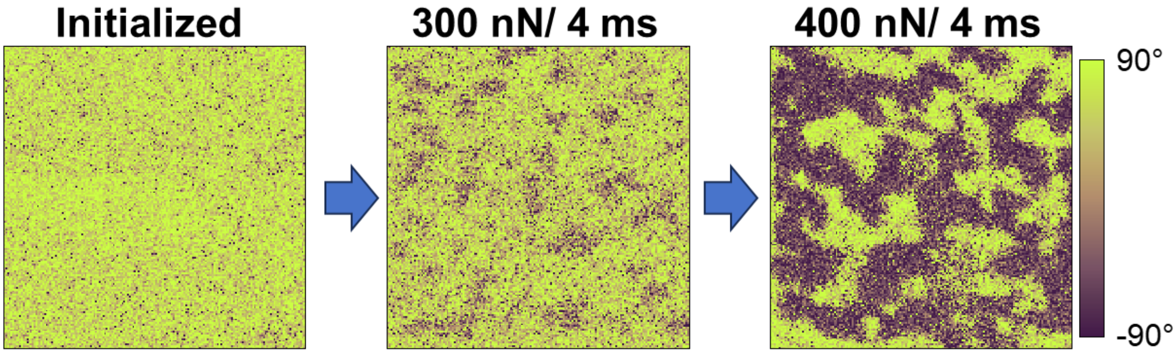

Supplementary Figure 6. PFM phase images of the 24 nm-thick CIPS on Ti obtained after initialization and after sequential force applications of 4 ms-long 300 nN and 400 nN. Size: 1 μm × 1 μm. In particular, a single 4 ms-long force pulse of 300 nN results in the formation of oppositely polarized domains, covering 18.7% of the area, confirming mechanical polarization switching. A stronger force of 400 nN further increases the switched domain area to 56.2%.

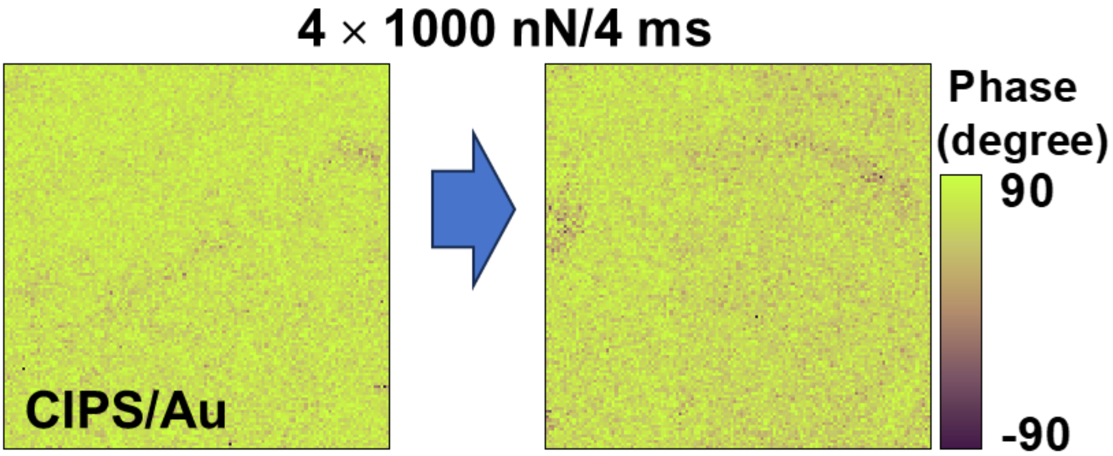

Supplementary Figure 7. PFM phase images of CIPS/Au obtained after electrical-erasing initialization and after four 4 ms-long force pulses of 1000 nN. Negligible polarization switching is achieved.

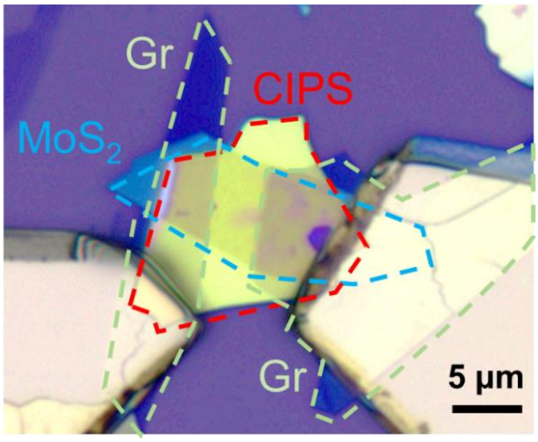

Supplementary Figure 8. Optical microscope image of the fabricated transistor presented in Fig.4c. The dashed lines provide the eye guides to the edges of each functional layers.

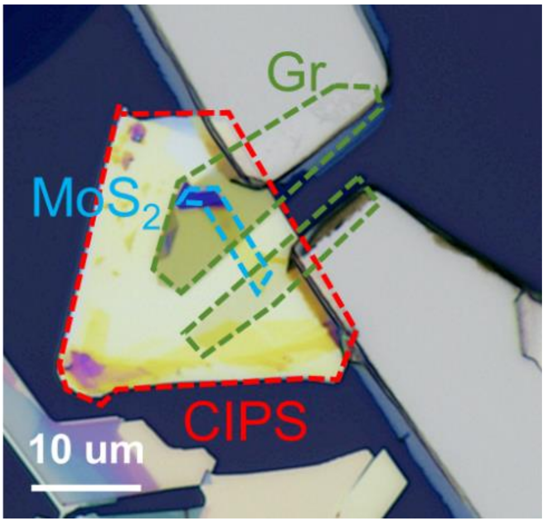

Supplementary Figure 9. Optical microscope image of the additional device fabricated and measured to verify the reproducibility of multi-level conductance states controlled by mechanical gate.

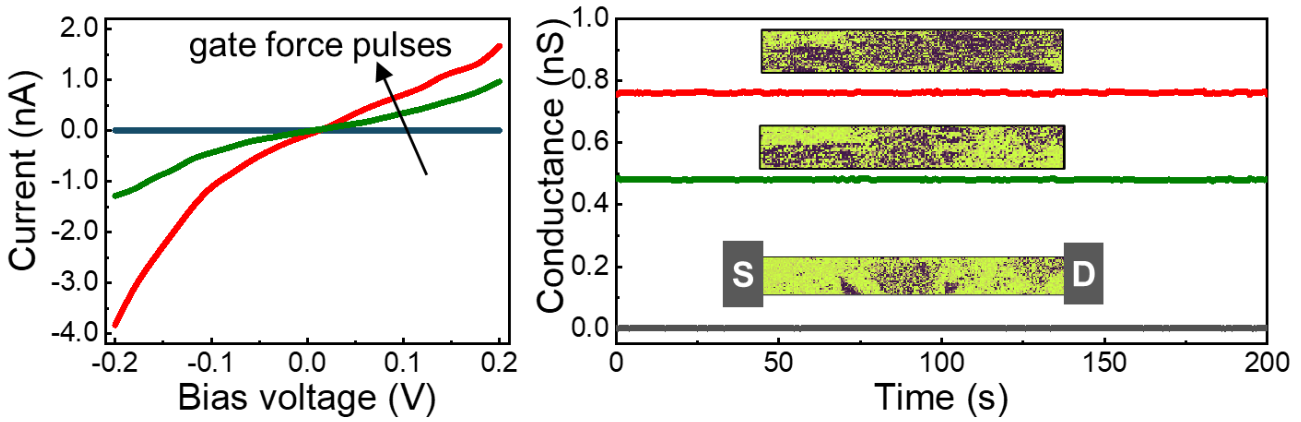

Supplementary Figure 10. Multi-level conductance behavior controlled by force pulses via the mechanical gate in the device shown in Fig. S9. The output curves demonstrate three distinct conductance states induced by sequential gate pulse operations. The retention characteristics of these states are maintained for at least 200 seconds. Insets show the corresponding PFM phase images of the gated CIPS region on the top the MoS_2 channel, as a rectangular area between graphene contacts along the current path. Gradual polarization switching is clearly observed after each gate force pulse application, supporting the reproducibility of the conductance states.

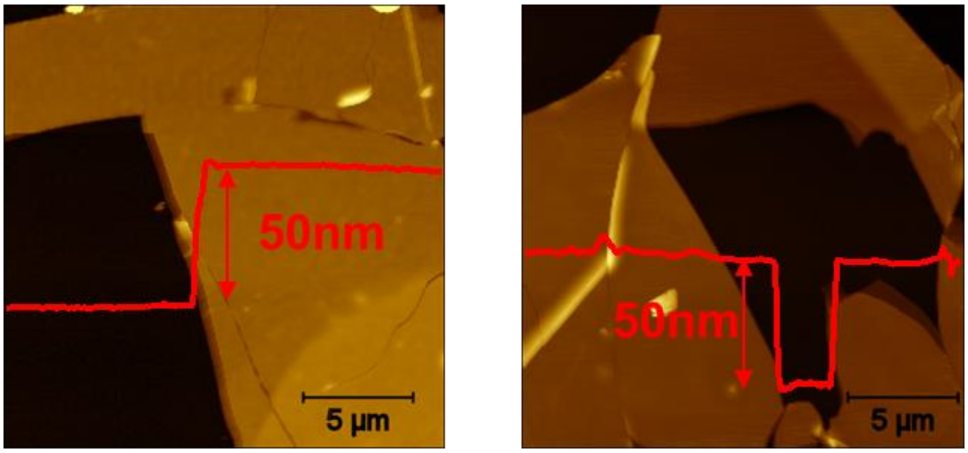

Supplementary Figure 11. AFM images of CIPS samples on different substrates, the line height profiles indicate the thicknesses of ~50 nm.

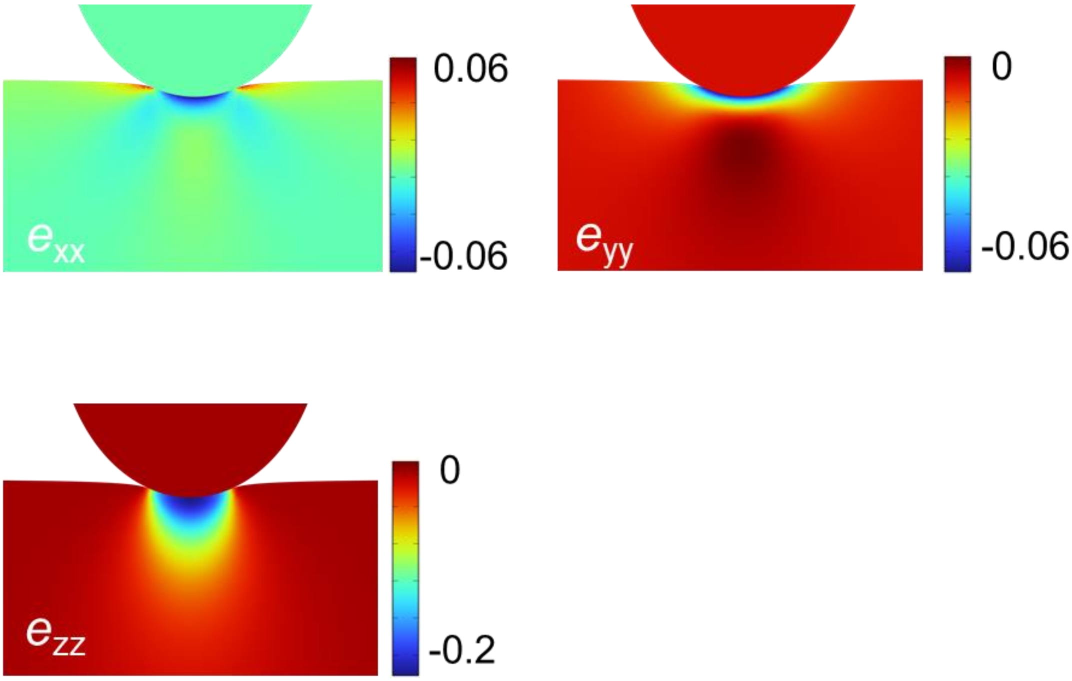

Supplementary Figure 12. Finite element simulation results of the distributions of e_{xx}, e_{yy} and e_{zz} in the deformed CIPS under tip force load of 600 N. e_{xx}, e_{yy} and e_{zz} are the strain distributions along various directions caused by the tip-induced deformation, which are dimensionless quantities representing relative deformation, *i.e.*, the ratio of the change in length to the initial length.

Supplementary Note 1.

Flexoelectricity depends on gradients of strain, the component of the induced polarization can be expressed as $P_i = f_{ijkl}\frac{\partial e_{kl}}{\partial x_j}$, where f_{ijkl} is flexoelectric coefficient tensor, e_{kl} is the strain tensor, x_j is the coordinate. Hence, the bound charge density is expressed as $\rho = -\nabla \cdot \boldsymbol{P}$. The resulting flexoelectric field $\boldsymbol{E}$ is therefore calculated through Gauss's law as $E = -\frac{\boldsymbol{P}}{\varepsilon_0 \varepsilon_\gamma}$ with $\varepsilon_0\varepsilon_\gamma$ being the dielectric constant of the ferroelectric. The flexoelectric voltage is then the line integral of the field, $\boldsymbol{V} = -\int \boldsymbol{E} \cdot \boldsymbol{dl}$. In the case of probe tip induced force on a thin film with out-of-plane polarization, only out-of-plane polarization P_z is considered and strain gradients are dominated by the out-of-plane direction z. Hence, all the lateral field component can be neglected. Flexoelectric coupling reduces to effective constants f_{33jj}, *i.e*, flexoelectric coefficient in z-direction from various strain components. In this analysis, it also assumes quasi-static conditions and a spatially homogeneous and isotropic medium, where the strain gradient-induced polarization varies smoothly in space. Under these assumptions, the induced flexoelectric field remains conservative and hence curl-free, allowing the use of a scalar potential. Then polarization can be simplified as $P_z = f_{3311}\frac{\partial e_{xx}}{\partial z} + f_{3322}\frac{\partial e_{yy}}{\partial z} + f_{3333}\frac{\partial e_{zz}}{\partial z}$. Subsequently, the flexoelectric field becomes $E_{\text{flexo}} = \frac{f_{3311}}{\varepsilon_0\varepsilon_\gamma}\frac{\partial e_{xx}}{\partial z} + \frac{f_{3322}}{\varepsilon_0\varepsilon_\gamma}\frac{\partial e_{yy}}{\partial z} + \frac{f_{3333}}{\varepsilon_0\varepsilon_\gamma}\frac{\partial e_{zz}}{\partial z}$. And finally, the flexoelectric voltage is expressed as $V_{\text{flexo}} = \frac{f_{3311}e_{xx} + f_{3322}e_{yy} + f_{3333}e_{zz}}{\varepsilon_0\varepsilon_\gamma}$.
